# A Failure Risk-Aware Multi-Hop Routing Protocol in LPWANs Using Deep Q-Network

**DOI:** 10.3390/s25144416

**Published:** 2025-07-15

**Authors:** Shaojun Tao, Hongying Tang, Jiang Wang, Baoqing Li

**Affiliations:** 1 Science and Technology on Micro-System Laboratory, Shanghai Institute of Microsystem and Information Technology, Chinese Academy of Sciences, Shanghai 200050, China; tsjun@mail.sim.ac.cn (S.T.); tanghy@mail.sim.ac.cn (H.T.); 2 University of Chinese Academy of Sciences, Beijing 100049, China

**Keywords:** routing failure risk value, deep Q-network, multi-hop routing, LPWANs

## Abstract

Multi-hop routing over low-power wide-area networks (LPWANs) has emerged as a promising technology for extending network coverage. However, existing protocols face high transmission disruption risks due to factors such as dynamic topology driven by stochastic events, dynamic link quality, and coverage holes induced by imbalanced energy consumption. To address this issue, we propose a failure risk-aware deep Q-network-based multi-hop routing (FRDR) protocol, aiming to reduce transmission disruption probability. First, we design a power regulation mechanism (PRM) that works in conjunction with pre-selection rules to optimize end-device node (EN) activations and candidate relay selection. Second, we introduce the concept of routing failure risk value (RFRV) to quantify the potential failure risk posed by each candidate next-hop EN, which correlates with its neighborhood state characteristics (i.e., the number of neighbors, the residual energy level, and link quality). Third, a deep Q-network (DQN)-based routing decision mechanism is proposed, where a multi-objective reward function incorporating RFRV, residual energy, distance to the gateway, and transmission hops is utilized to determine the optimal next-hop. Simulation results demonstrate that FRDR outperforms existing protocols in terms of packet delivery rate and network lifetime while maintaining comparable transmission delay.

## 1. Introduction

Multi-hop routing in low-power wide-area networks (LPWANs) has emerged as a promising solution for expanding geographical coverage [1,2]. Within such networks, event-driven architectures are widely adopted to enhance energy efficiency [3,4]. However, multi-hop routing over event-driven LPWANs is challenged by high transmission disruption risk. Specifically, dynamic link quality introduces unstable link connections, while imbalanced energy consumption and nonuniform end-device node (EN) distribution lead to coverage holes that disrupt data forwarding [5,6,7]. Consequently, developing multi-hop routing protocols that guide ENs to select routes with low disruption probability is critical.

Over the past decades, numerous multi-hop routing protocols have been proposed to determine optimal relays by evaluating the intrinsic EN state and neighborhood state [8,9,10,11,12]. However, these studies primarily focus on assessing link quality within the neighborhood state. By overlooking the number and residual energy of neighbors, these methods struggle to avoid selecting ENs that introduce high routing failure risk. Specifically, ENs with few neighbors exhibit higher transmission failure probabilities due to limited next-hop availability, while those connected to low-energy neighbors are prone to instability caused by energy depletion during data forwarding. Therefore, developing a comprehensive neighborhood state assessment framework to avoid relays that introduce high routing failure risk is imperative.

Given these, we propose a failure risk-aware deep Q-network-based multi-hop routing (FRDR) protocol. In FRDR, by evaluating multiple neighborhood state characteristics, a distinct routing failure risk value (RFRV) is assigned to each EN. RFRV is then integrated with other metrics into the reward function of a deep Q-network (DQN)-based routing decision framework to determine the optimal next-hop. The DQN employs reinforcement learning (RL), where agents continuously interact with external environments to learn optimal policies that maximize cumulative rewards [13]. Furthermore, by employing deep neural networks (DNNs) to approximate the Q-function within the Q-learning framework, DQN can effectively handle multi-hop routing under dynamic and complex conditions [14,15].

The main contributions of our study are summarized as follows:

- We design a novel power regulation mechanism (PRM) that adaptively adjusts activation ranges based on the average signal-to-noise ratio (SNR) of received signals from neighbors. This mechanism further incorporates pre-selection rules to optimize EN activations and candidate relay selection.
- We introduce the concept of routing failure risk value (RFRV) to quantify the potential failure risk posed by each candidate next-hop EN, which is evaluated based on its neighborhood state characteristics, including the number of neighbors, residual energy level, and link quality.
- We develop a DQN-based routing decision mechanism that integrates RFRV into the reward function. Building upon metrics such as residual energy, distance to the gateway, and transmission hop count, our mechanism prioritizes low-RFRV ENs, thereby reducing transmission failures.
- Through meticulous evaluation across various metrics, our simulation results demonstrate the advantages of FRDR in improving packet delivery rate and network lifetime while maintaining comparable transmission delay.

The remainder of this paper is organized as follows. Related studies are discussed in Section 2. Section 3 presents a brief review of DQN, and Section 4 introduces system models. In Section 5, the details of FRDR are described. Simulation results are thoroughly analyzed in Section 6 to illustrate the superiority of FRDR over other protocols, while Section 7 concludes this paper.

## 2. Related Studies

Over the past decades, numerous multi-hop routing protocols have been investigated, with a focus on relay selection strategies to optimize routing performance. In [8], link state information within two hops was considered when selecting relays to minimize delay and reduce packet loss. However, this two-hop dependency incurs high computational overhead in dynamic networks with frequent topology changes. In [9], the candidate relay with the highest reliability was selected to establish high-reliability and low-latency routes. Nevertheless, due to the dependence on predefined fuzzy rules, its adaptability to unmodeled network scenarios is limited. A method based on link quality prediction was proposed in [10], where a fuzzy logic system that incorporates distance, residual energy, and link quality (estimated via Kalman filtering) was adopted in relay decisions. However, this method is susceptible to model mismatch in event-driven networks, as bursty traffic violates the Markovian assumption underlying Kalman filtering-based prediction.

Given the limitations of traditional approaches, RL-based methods have emerged as a promising solution. These methods enable agents to learn optimal routing policies through real-time interaction with the external environment and reward-driven optimization, eliminating dependence on predefined models [13,14]. A Q-learning-based routing protocol was developed in [16], where energy consumption, bandwidth utilization, throughput, and data latency are jointly considered during relay selection. Similarly, ref. [17] proposed a Q-learning framework to reduce packet losses by deprioritizing predicted faulty nodes within routing decisions. In [15], a DQN-based intelligent routing (DQIR) protocol that balances residual energy distribution while minimizing routing distance was introduced to select relays. To address challenges such as insufficient adaptability to network topology changes, high communication delays, and short network lifetime in multi-hop routing, a dueling double deep Q-network was employed in [14] to optimize routing decisions. In [18], a reinforcement learning framework that integrates different node centrality metrics was developed to optimize relay selection.

A review of existing research reveals that while neighborhood state has been incorporated into relay selection decisions, these studies primarily focus on link quality without simultaneously considering the number of neighbors and their residual energy. This narrow focus prevents these methods from effectively excluding relays that introduce high routing failure risk, particularly in dynamic and complex networks. Geared toward this shortcoming, we propose FRDR in this article.

## 3. Brief Review of DQN

An RL framework is typically modeled as a Markov Decision Process (MDP), characterized by a tuple $\langle S,A,P,R\rangle$, where S represents the state space, A denotes the action space, P is the state-transition probability, and R signifies the rewards. At each time step t, the agent executes the action $a_{t}\in A$ determined by the policy π based on the current state $s_{t}\in S$. Subsequently, the environment provides the agent with an immediate reward $r_{t}\in R$ contingent upon $a_{t}$ and transitions to the next state $s_{t+1}$. This process generates an experience $\left(s_{t},a_{t},r_{t},s_{t+1}\right)$. The overarching goal of the agent is to derive an optimal policy $\pi^{*}$ that maximizes the expected cumulative reward, thereby optimizing long-term performance within the given MDP framework [15].

Q-learning is a value-based RL algorithm that iteratively refines policies to approximate $\pi^{*}$. The action-value function $Q\left(s,a\right)$ estimates the expected return of taking action a in state s, which is updated iteratively using the following formula:

(1) $$Q\left(s,a\right)\leftarrow Q\left(s,a\right)+\alpha \left\{r+\gamma \underset{a^{\prime }\in A}{\max}Q\left(s^{\prime },a^{\prime }\right)-Q\left(s,a\right)\right\},$$

where $\underset{a^{\prime }\in A}{\max}Q\left(s^{\prime },a^{\prime }\right)$ is the maximum Q-value over all possible actions $a^{\prime }$ in the subsequent state $s^{\prime }$, γ is the discount factor, and α is the learning rate. $\pi^{*}$ directs the agent toward actions that yield the highest $Q\left(s,a\right)$ in each state.

When the state space is large, exhaustively computing $Q\left(s,a\right)$ becomes infeasible. Consequently, DQN is adopted to approximate $Q\left(s,a\right)$, where the output is $Q\left(s,a;\omega \right)\approx Q\left(s,a\right)$. Here, ω represents the weights of the DNN, and the stochastic gradient descent (SGD) algorithm is used to update parameters.

However, the neural network can become unstable owing to correlations between Q-value and target value, or small updates to Q-value at each step. To address this instability, experience replay and a quasi-static target network are employed in DQN [18]. In experience replay, at each time step t, an experience sample $e_{t}=\left(s_{t},a_{t},r_{t},s_{t+1}\right)$ is stored in a replay memory $M=\left\{e_{1},e_{2},\cdots ,e_{t}\right\}$. During training, the agent randomly samples a minibatch of experiences from M, thus removing the correlations between continuous samples and improving the stability and efficiency of learning. Additionally, an independent target neural network with weights $\omega^{-}$ is used for the quasi-static target network. The loss function $L\left(\omega \right)$ is calculated as follows:

(2) $$L\left(\omega \right)=E_{\left(s,a,r,s^{\prime }\right)\in M}\left[{\left(y\left(s^{\prime },r\right)-Q\left(s,a;\omega \right)\right)}^{2}\right],$$

where $y\left(s^{\prime },r\right)$ is the output of the target neural network:

(3) $$y\left(s^{\prime },r\right)=r+\gamma \underset{a^{\prime }\in A}{\max}Q\left(s^{\prime },a^{\prime };\omega^{-}\right),$$

where $\omega^{-}$ is synchronized with ω every C steps. This approach decouples the target value computation from the Q-network weights, thereby reducing the likelihood of divergence and ensuring more stable learning.

## 4. System Models

### 4.1. Network Model

Without loss of generality, we consider a network where N ENs are randomly distributed within an L×L monitoring area with nonuniform density. As established in [19,20,21,22], the network model satisfies the following assumptions to construct a standardized scenario:

1. A gateway (GW) is located in the center of the network and remains powered on. Central placement simplifies the network model, providing a consistent reference point for all ENs while facilitating a more balanced distribution of data flow.
2. All ENs are homogeneous. This configuration minimizes performance variations due to hardware differences, thereby facilitating an unbiased evaluation of the logic and effectiveness of routing protocols under consistent operating parameters.
3. Both ENs and GW are stationary after deployment. This configuration eliminates route fluctuations caused by the mobility of ENs and GW.
4. All ENs are synchronized and can determine their locations via Global Positioning System (GPS) or other self-localization algorithms. Synchronization is essential for ordering control and data packets in negotiation-based protocols, while geographic information is fundamental for distance-based relay selection.
5. The links are symmetric. This assumption ensures bidirectional connectivity and consistent link characteristics, thereby avoiding complications from unidirectional paths that disrupt acknowledgment-dependent routing protocols.

### 4.2. Routing Failure Risk Value

**Figure 1:**
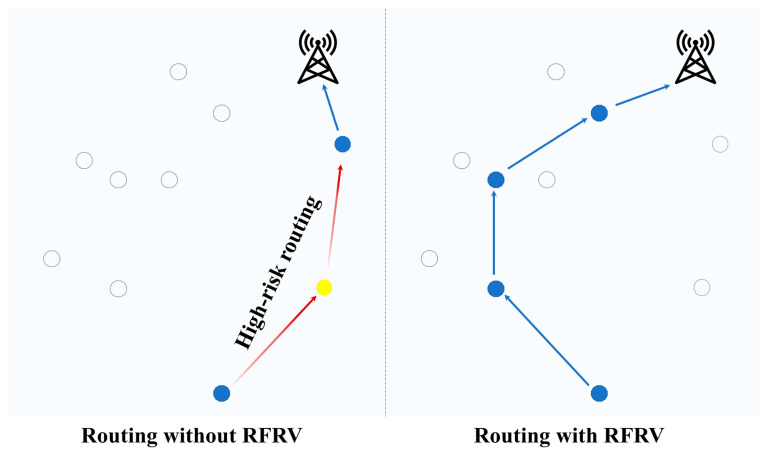
Routing diagram with and without RFRV.

To reduce transmission disruption probability, ENs with higher Routing Failure Risk Value (RFRV) are deprioritized in FRDR. The effectiveness of this approach is demonstrated in Figure 1.

**Definition**  **1.** *For a given EN, its neighboring ENs are all ENs located within its maximum direct communication range*.

Generally, neighborhood state characteristics, including the number of neighbors $N_{n}$, the residual energy level of neighbors $E_{n}$, and link quality LQ, are jointly considered when evaluating RFRV. An EN with lower $N_{n}$, $E_{n}$, and LQ is associated with a higher RFRV. For $EN_{i}$, $RFRV_{i}$ can be computed via Equations (4)–(6).

(4) $$RFRV_{i}=\lambda_{1}{\tilde{N}}_{n}^{i}+\lambda_{2}{\tilde{LQ}}_{i}+\lambda_{3}{\tilde{E}}_{n}^{i},$$

(5) $$LQ_{i}=\sqrt{\frac{\bar{RSSI_{i}}-RSSI_{th}}{RSSI_{th}}\cdot \frac{\bar{SNR_{i}}-SNR_{th}}{SNR_{th}}},$$

(6) $$E_{n}^{i}=\bar{e_{res}^{n}\left(i\right)}/e_{init},$$

(7) $$\tilde{x}=\left(x-x_{\min}\right)/\left(x_{\max}-x_{\min}\right),$$

where $\bar{RSSI_{i}}$ and $\bar{SNR_{i}}$ denote the average received signal strength indicator (RSSI) and signal-to-noise ratio (SNR) at $EN_{i}$ from signals transmitted by its neighbors, while $RSSI_{th}$ and $SNR_{th}$ are the corresponding thresholds. $\bar{e_{res}^{n}\left(i\right)}$ indicates the average residual energy of neighbors, while $e_{init}$ is the initial energy. To eliminate dimensional differences among heterogeneous indicators, $N_{n}$, $E_{n}$, and LQ are standardized by the min-max normalization, as defined in Equation (7).

The weights $\lambda_{i}\left(i=1,2,3\right)$ in Equation (4) are determined using the Analytical Hierarchy Process (AHP) [23]. Benefiting from its capability in establishing quantitative frameworks for complex and ambiguous decision-making problems, as well as systematically relating criterion weights to overarching objectives, AHP is widely adopted for deriving criterion weights in multi-criteria decision analysis [24].

The AHP process begins by constructing a pairwise comparison matrix A for decision criteria, as defined in Equation (8).

(8) $$A={\left(a_{ij}\right)}_{k\times k}=\left[\begin{array}{cccc} 1 & a_{12} & \ldots & a_{1k}\\ a_{21} & 1 & \ldots & a_{2k}\\ \vdots & \vdots & \vdots & \vdots \\ a_{k1} & a_{k2} & \ldots & 1 \end{array}\right],$$

where each element $a_{ij}$ denotes the relative importance of the criterion associated with row index i compared to the criterion related to the column index j. When constructing A, a 1–9 scale [25] is widely adopted to quantify the relative importance between each pair of criteria. This well-known AHP scale is shown in Table 1.

The weight vector w is then derived by solving the characteristic equation:

(9) $$Aw=\lambda_{\max}w,$$

where $\lambda_{\max}$ is the largest eigenvalue of the pairwise comparison matrix A.

Since w represents unnormalized priorities, the final criteria weights $w_{i}^{\prime }$ are obtained through normalization:

(10) $$w_{i}^{\prime }=w_{i}/\sum_{j=1}^{k}w_{j},$$

where k is the order of A (i.e., the number of criteria).

Since pairwise comparisons in AHP are heavily dependent on human judgment, they are susceptible to inconsistencies. To address this issue, a standard procedure is provided in [26] to check for the consistency of the pairwise comparison matrix by utilizing the largest eigenvalue $\lambda_{\max}$. The deviation of $\lambda_{\max}$ from the matrix dimension k is quantified by the Consistency Index (CI):

(11) $$CI=\frac{\lambda_{\max}-k}{k-1},$$

To benchmark CI, Random CI (RI) is also proposed in [26] (Table 2), which is derived from randomly generated reciprocal matrices of various dimensions. The Consistency Ratio (CR) is then calculated as follows:

(12) $$CR=\frac{CI}{RI},$$

According to the established threshold [27], CR≤0.1 indicates that the results are satisfactory. Otherwise, the pairwise comparison matrix must be re-evaluated.

According to Equations (8)–(10), the weights $\lambda_{i}\left(i=1,2,3\right)$ in Equation (4) are determined using the pairwise comparison matrix presented in Table 3. This matrix satisfies the consistency requirement (CR = 0.0079 < 0.1), confirming the reliability of the weight results.

## 5. Detailed Description of the Proposed Protocol

**Figure 2:**
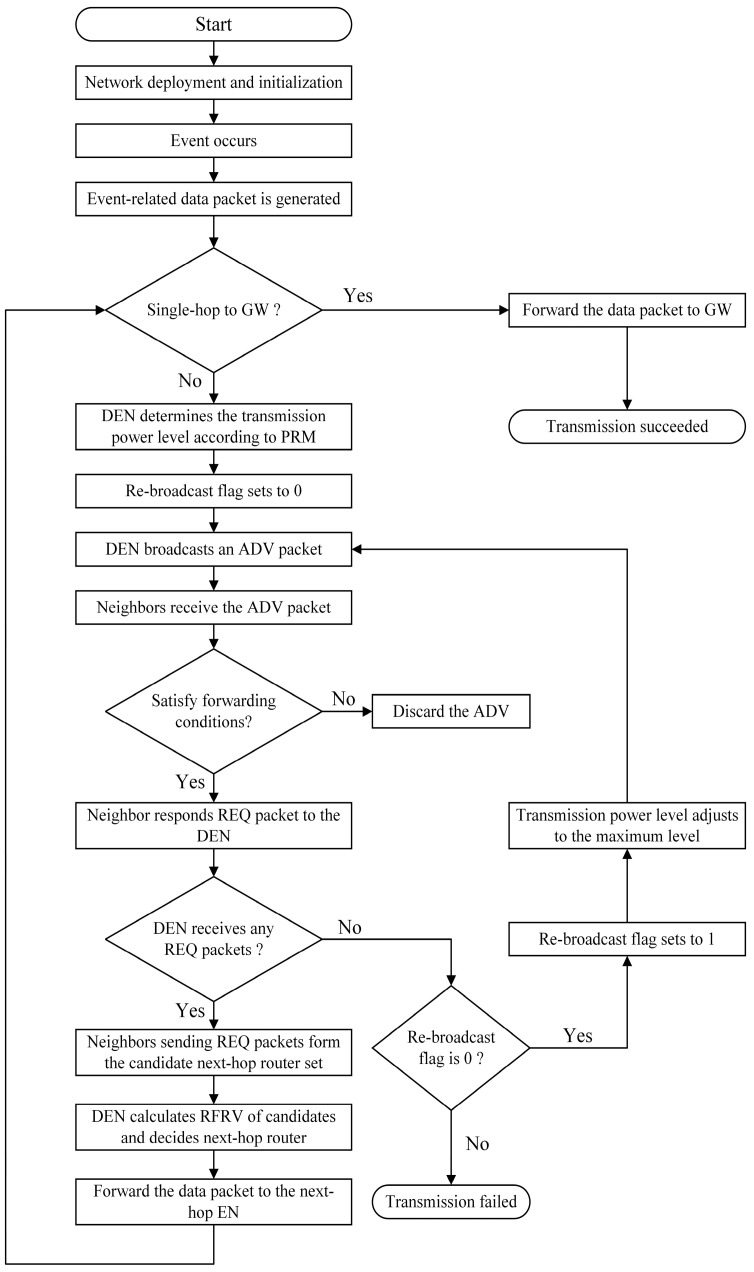
Overall flowchart of FRDR.

In this section, FRDR is introduced in detail with the overall flowchart illustrated in Figure 2.

To deliver an event-related data packet to the GW, data-holding EN (DEN) in FRDR first checks whether the GW is within its one-hop communication range. If direct transmission is feasible, the data packet is forwarded directly. Otherwise, DEN triggers the relay selection process, which consists of two phases: candidate router selection and optimal relay decision-making.

**Figure 3:**
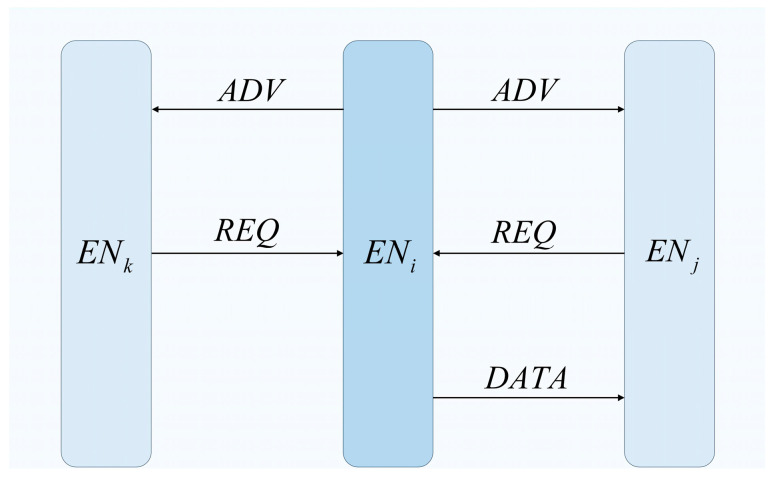
Diagram of SPIN-based message interaction flow.

Given the need for real-time topology awareness in dynamic networks, FRDR utilizes the Sensor Protocol for Information via Negotiation (SPIN) [28] to manage neighbor discovery and state updates, with its message sequence detailed in Figure 3. The specific workflow is outlined as follows:

DEN initiates the relay selection process by broadcasting an Advertisement (ADV) message with transmission power regulated by the Power Regulation Mechanism (PRM). This mechanism dynamically adjusts EN activation ranges based on the average neighbor signal SNR to enhance energy efficiency. Neighboring ENs that receive the ADV message parse its metadata to extract information such as the DEN-to-GW distance.

To prevent redundant participation, a pre-selection mechanism is employed to restrict relay requests from ENs that receive the ADV message. Each EN autonomously determines whether to apply for packet forwarding based on its residual energy and distance to the GW. Eligibility criteria for the application are as follows:

(1) The distance from the EN to the GW must be shorter than the DEN-to-GW distance recorded in the ADV metadata, thereby preventing data backhaul.
(2) The residual energy of the EN must exceed a predefined threshold $e_{th}$, which is derived from the energy cost of receiving and forwarding a data packet at the minimum transmission power level. This criterion prevents resource wastage caused by ENs with insufficient energy applying for relay tasks.

Neighboring ENs that fail either condition discard the ADV packet, and qualified ENs respond with a Request (REQ) message containing self-reported metrics (i.e., residual energy and distance to the GW). These responding ENs form the candidate set of next-hop routers.

If the DEN receives no REQ packet within the designated reception window, it rebroadcasts the ADV packet at its maximum power level to activate more potential relays. If this second broadcast also fails to elicit any response, the transmission is deemed a failure due to the unavailability of suitable relays.

Conversely, when REQ responses are received, the DEN executes a DQN-based routing decision mechanism, as detailed in Section 5.2, to determine the optimal relay from candidates. Following this selection, the DEN forwards the data packet to the chosen relay. Upon completing the role transition, the current DEN exits the routing process and enters a low-power sleep mode, where it awaits its next activation to minimize energy consumption.

### 5.1. Power Regulation Mechanism

By default, ENs operate at maximum power to maintain periodic neighbor information exchange. However, this configuration becomes inefficient during data transmission, as excessive power causes resource wastage (e.g., redundant EN activations). To address this issue, FRDR introduces a Power Regulation Mechanism (PRM) that adaptively adjusts transmission power levels based on the average SNR of received signals from neighbors, thereby reducing overhead.

As detailed in Algorithm 1, the standard Adaptive Data Rate (ADR) algorithm [29] adjusts data rates based on SNR to optimize throughput and energy efficiency.
**Algorithm 1.** Standard Adaptive Data Rate Algorithm.**Initialize:**$\;\mathrm{Spreading}\;\mathrm{factor}\;SF\in \left[7,12\right]$$,\;\mathrm{Transmitting}\;\mathrm{Power}\;TP\in \left[2\;\mathrm{dBm},14\;\mathrm{dBm}\right]$1:$SNR_{req}$
← demodulation floor (current data rate)2:$SNR_{\max}$
← max (SNR of last 20 frames)3:$SNR_{margin}$$\;\leftarrow \;SNR_{\max}-SNR_{req}-Margin\_dB$4:NStep$\;\leftarrow \;\mathrm{int}(SNR_{\max}/3$
)5:while NStep>0$\;\mathrm{and}\;SF>SF_{\min}\;$**do**6:  SF ← SF−17:  NStep ← NStep−18:**end while**9:while NStep>0$\;\mathrm{and}\;TP>TP_{\min}\;$**do**10:  TP ← TP−311:  NStep ← NStep−112:**end while**13:while NStep<0$\;\mathrm{and}\;TP<TP_{\max}\;$**do**14:  TP ← TP+315:  NStep ← NStep+116:**end while**17:Output: TP and SF

Building upon the standard ADR framework, we propose the PRM, which redirects optimization from data rates to transmission power levels. The workflow of PRM operates as follows:

Step 1: Calculate the average SNR $SNR_{avg}$ of the most recently received signals transmitted by n neighbors.

Step 2: Subtract a predefined margin Ma (default: 10 dB) from the difference between $SNR_{avg}$ and $SNR_{th}$ to determine the SNR margin $SNR_{margin}$, i.e., $SNR_{margin}=\left(SNR_{avg}-SNR_{th}\right)-Ma$.

Step 3: Adjust the current transmission power level $l_{tx}$ based on $SNR_{margin}$. If $SNR_{margin}>0$, it suggests that $l_{tx}$ can be reduced without compromising communication reliability.

Given the dynamic nature of the link environment, relying on a fixed neighbor count, n, may lead to inaccurate decisions. To overcome this limitation, PRM adaptively adjusts n based on link variability. Specifically, the DEN randomly selects n neighbors from its routing table and calculates the volatility rate of the link environment, $R_{change}$, using Equation (13).

(13) $$R_{change}=\frac{1}{n}\sum_{i=1}^{n}\frac{\left|RSSI_{\mathrm{SL}}\left(i\right)-RSSI_{L}\left(i\right)\right|}{\left|RSSI_{th}-RSSI_{L}\left(i\right)\right|},$$

where $RSSI_{L}\left(i\right)$ and $RSSI_{SL}\left(i\right)$ denote the RSSI of the last and penultimate signals received by DEN from the i-th EN, respectively.

Higher values of $R_{change}$ indicate a more volatile link environment. When $R_{change}$ exceeds the threshold $R_{change}^{-}$, to enhance decision accuracy, PRM increases n by 1 to incorporate diverse information from additional neighbors into the decision-making process. This adjustment repeats until either $R_{change}\leq R_{change}^{-}$ or $n\geq N_{n}$, thereby achieving an adaptive balance between decision accuracy and computational overhead. The initial empirical value of n is set at 3 according to [29,30]. The detailed PRM workflow is described in Algorithm 2.
**Algorithm 2.** Power Regulation Mechanism**Input:** $SNR_{th}$$,\;\mathrm{the}\;\mathrm{upper}\;\mathrm{limit}\;l_{tx}^{\max}$$\;\mathrm{and}\;\mathrm{lower}\;\mathrm{limit}\;l_{tx}^{\min}$$\;\mathrm{of}\;l_{tx}$$\;(\mathrm{with}\;\mathrm{initial}\;\mathrm{value}\;l_{tx}=l_{tx}^{\max}$$),\;\mathrm{SNR}\;\mathrm{and}\;\mathrm{RSSI}\;\mathrm{of}\;\mathrm{received}\;\mathrm{signals}\;\mathrm{from}\;\mathrm{neighbors},\;\mathrm{the}\;\mathrm{number}\;\mathrm{of}\;\mathrm{neighbors}\;N_{n}$$\mathrm{Output}\text{:}\;l_{tx}$1:Randomly select
 n 
$\mathrm{neighbors}\;\mathrm{and}\;\mathrm{calculate}\;R_{change}$
by Equation (13)2:$\mathrm{if}\;N_{n}\leq 3\;$**then**3:$\;\;\;l_{tx}=l_{tx}^{\max}$4:**else**5:$\;\;\;\mathrm{if}\;R_{change}>R_{change}^{-}$$\;\mathrm{and}\;n<N_{n}\;$**then**6:       n=n+17:      Go to line 18:**   end if**9:**end if**10:$\mathrm{calculate}\;SNR_{avg}$$\;\mathrm{and}\;SNR_{margin}$11:$NStep\leftarrow round\left(SNR_{margin}/3\right)$12:while NStep>0$\;\mathrm{and}\;l_{tx}>l_{tx}^{\min}\;$**do**13:$\;\;\;l_{tx}=l_{tx}-1$ and NStep=NStep−114:**end while**

### 5.2. DQN-Based Routing Decision Mechanism

At time step t, all ENs that respond to a REQ packet form the candidate relay set ${\mathbb{N}}_{t}^{cf}$, from which DEN selects the optimal next-hop router. However, an EN selected as a relay will consume more energy, which may lead to unbalanced energy distribution across the network and potentially cause coverage holes. Furthermore, selecting candidates with high RFRV increases transmission disruption probability. Given these, we propose a low-latency, long-lifetime, and high-success-rate routing decision mechanism. The optimal relay selection is formulated as follows:

(14) $$\begin{array}{cc} \max & e_{res}\left(i,t\right)\\ \min & RFRV_{i,t}\\ \min & dst_{i}\\ \min & hop\\ s.t. & EN_{i}\in {\mathbb{N}}_{t}^{cf} \end{array}$$

where $e_{res}\left(i,t\right)$ denotes the residual energy of $EN_{i}$ at time step t, $dst_{i}$ is the distance between $EN_{i}$ and the GW, and hop is the packet transmission hop count.

#### 5.2.1. MDP Model for FRDR

**Figure 4:**
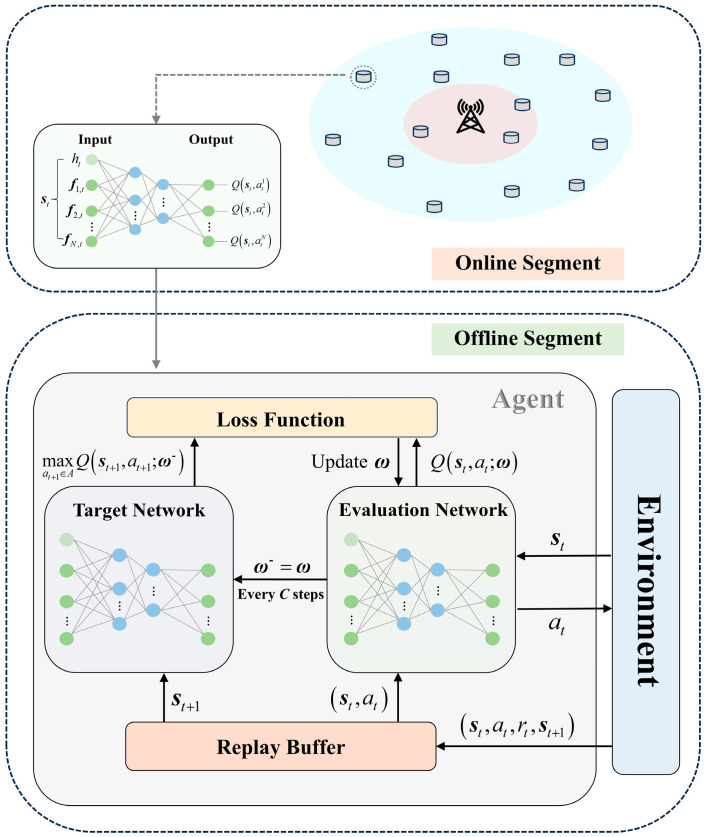
Framework of DQN-based routing decision mechanism.

Given the dynamic nature of network conditions, the routing decision process in FRDR is modeled as an MDP and solved using DQN. The overall framework is illustrated in Figure 4.

By modeling DEN as an agent, the corresponding states, actions, and reward functions are defined as follows:

- States: The state integrates hop count and features of EN to form a unified vector $s_{t}\in {\mathbb{R}}^{3N+1}$. To handle dynamic fluctuations in the number of candidate relays, FRDR employs a feature masking mechanism. For each $EN_{i}$ at time step t, its feature vector is defined as follows:
  (15) $$f_{i,t}=\left\{\begin{array}{cc} \left[dst_{i}/L,e_{res}\left(i,t\right)/e_{init},RFRV_{i,t}\right], & EN_{i}\in {\mathbb{N}}_{t}^{cf}\\ \left[1,0,1\right], & otherwise \end{array}\right.$$
  Based on the current hop count $h_{t}$, the overall state $s_{t}$ is expressed as follows:
  (16) $$s_{t}=\left\{\begin{array}{cc} {\left[h_{t},{⊕}_{i=1}^{N}\left[1,0,1\right]\right]}^{T}, & failure\\ {\left[h_{t},{⊕}_{i=1}^{N}\left[0,1,0\right]\right]}^{T}, & success\\ {\left[h_{t},{⊕}_{i=1}^{N}f_{i,t}\right]}^{T}, & intermediate \end{array}\right.$$
  where ⊕ denotes vector concatenation.
- Actions: By executing action $a_{t}$ at time step t, the agent selects the corresponding EN as the next-hop router, i.e., $a_{t}=i\left(i\in \left\{1,2,\ldots ,N\right\}\right)$ indicates that $EN_{i}$ is chosen as the relay.
- Reward Function: To determine the optimal relay in Equation (14), the reward function in FRDR is designed to guide the agent towards solutions that maximize residual energy, minimize RFRV, reduce distance to the GW, and minimize hop count. It is defined as follows:
  (17) $$r_{t}=\left\{\begin{array}{cc} R_{\max}, & C_{1}\\ -R_{\max}, & C_{2}\\ {\hat{r}}_{t}, & C_{3} \end{array}\right.$$
  In Equation (17), $C_{1}$ represents successful packet delivery to the GW, for which a positive reward $R_{\max}$ is granted. Conversely, $C_{2}$ denotes transmission disruption, which is penalized with $-R_{\max}$. All other cases fall under $C_{3}$, where the composite reward ${\hat{r}}_{t}$ implements the optimization objectives from Equation (14) through specific reward components:
  (18) $$r_{t}^{1}=dst_{i}/L,$$
  (19) $$r_{t}^{2}=e_{res}\left(i,t\right)/e_{init},$$
  (20) $$r_{t}^{3}=RFRV_{i,t},$$
  (21) $$r_{t}^{4}=h_{t}/H_{\max},$$
  Note that $r_{t}^{1}$, $r_{t}^{2}$, and $r_{t}^{3}$ pertain to the attributes of ENs, while $r_{t}^{4}$ is a path attribute. By integrating Equations (18)–(21), the composite reward ${\hat{r}}_{t}$ is derived as Equation (23). Here, ${\tilde{r}}_{t}^{1}$ and ${\tilde{r}}_{t}^{3}$ are the normalized versions of $r_{t}^{1}$ and $r_{t}^{3}$ via Equation (7), while $r_{t}^{2}$ is normalized using Equation (22).
  (22) $$\tilde{x}=\left(x_{\max}-x\right)/\left(x_{\max}-x_{\min}\right),$$
  (23) $${\hat{r}}_{t}=\sum_{i=1}^{3}\mu_{i}\cdot {\tilde{r}}_{t}^{i}-\eta \cdot r_{t}^{4},$$
  The weights $\mu_{i}$ in Equation (23) are calculated via AHP using Equations (8)–(10). The pairwise comparison matrix for decision criteria in Equation (23) is presented in Table 4, with a CR of 0.0158. This CR value is well below the threshold of 0.1, confirming the logical coherence of the pairwise comparisons and the reliability of the weight results.

#### 5.2.2. DQN Architecture

The DQN architecture implemented in FRDR is detailed as follows:

1. Input layer: A fully connected (FC) layer is used as the input layer. The input feature dimension is set to 3N+1, corresponding to the pre-masked state $s_{t}$.
2. Hidden layer: The hidden layer comprises two FC layers with 64 and 32 neurons, respectively. For each FC layer, the Leaky Rectified Linear Unit (Leaky ReLU) activation function with a negative slope coefficient of 0.01 is employed. Moreover, the backpropagation gradients from non-candidate ENs are set to zero.
3. Output layer: We define an FC layer with N neurons as the output layer to generate raw Q-values $Q_{raw}$, where a linear activation function is utilized. Feature-based masking is then applied to compute final values by Equation (24).
  (24) $$Q=\left\{\begin{array}{ll} Q_{raw}, & EN_{i}\in {\mathbb{N}}_{t}^{cf}\\ -10^{5}, & otherwise \end{array}\right.$$

#### 5.2.3. Network Training and Routing Decision

At time step t, the agent gathers state information from candidate relays and constructs the state vector $s_{t}$ according to Equation (16). $s_{t}$ is fed into the DQN, which outputs Q-values corresponding to each EN. The agent then selects action $a_{t}$ using an annealing ε-greedy strategy, where ε decays as follows [31]:

(25) $$\varepsilon =\varepsilon_{end}+\left(\varepsilon_{start}-\varepsilon_{end}\right)\cdot \exp\left(-\tau \cdot N_{eps}^{now}\right),$$

where $\varepsilon_{start}$ and $\varepsilon_{end}$ represent the initial and terminal values of ε, respectively. $N_{eps}^{now}$ denotes the number of current training iterations, while τ is the attenuation rate. With probability 1−ε, the agent exploits by selecting the EN associated with the maximum Q-value. During exploration, a Weighted Probability Selection method that prioritizes candidates with lower RFRV is employed to enhance efficiency. The selection probability $p\varepsilon_{i}$ for $EN_{i}$ during exploration is given by:

(26) $$p\varepsilon_{i}=\left\{\begin{array}{cc} 1-\frac{RFRV_{i}}{\sum_{EN_{i}\in {\mathbb{N}}_{t}^{cf}}RFRV_{i}}, & EN_{i}\in {\mathbb{N}}_{t}^{cf}\\ 0, & otherwise \end{array}\right.$$

Upon determining $a_{t}$, DEN forwards the data packet to the corresponding EN. At the next time step, the state transitions to $s_{t+1}$, and the environment provides the agent with reward $r_{t}$ computed via Equation (17). The agent then constructs the transition tuple $\left(s_{t},a_{t},r_{t},s_{t+1}\right)$ and stores it in replay memory M. When M accumulates a sufficient number of samples, the agent randomly samples a minibatch from M every $C_{\exp}$ timesteps to train the DQN by minimizing the loss $\bar{L}\left(\omega \right)$ defined in Equation (2). Additionally, the target network is periodically synchronized with the evaluation network every $C_{t}$ timesteps.

The overall framework of the DQN-based routing decision mechanism is illustrated in Algorithm 3. Notably, the learning process is conducted in the virtual environment configured on computers to avoid high-performance demands on EN entities.
**Algorithm 3.** DQN-Based Routing Decision Mechanism$\mathrm{Input}\text{:}\;\varepsilon_{start}$$,\;\varepsilon_{end}$, τ, γ, α$,\;\mathrm{experience}\;\mathrm{replay}\;\mathrm{update}\;\mathrm{frequency}\;C_{\exp}$$,\;\mathrm{target}\;\mathrm{update}\;\mathrm{frequency}\;C_{t}$, minibatch size B$,\;\mathrm{maximum}\;\mathrm{training}\;\mathrm{episodes}\;N_{eps}^{\max}$$,\;\mathrm{maximum}\;\mathrm{iterations}\;N_{iter}^{\max}$**Initialize:** replay memory ***M***
$,\;\mathrm{experience}\;\mathrm{counter}\;N_{\exp}=0$
, evaluation network with random weights ω
$,\;\mathrm{target}\;\mathrm{network}\;\mathrm{with}\;\mathrm{weights}\;\omega^{-}=\omega$
**Offline Learning**1:$\mathrm{for}\;N_{eps}$$=1:N_{eps}^{\max}\;$**do**2: 
$\mathrm{for}\;N_{iter}$
$=1:N_{iter}^{\max}\;$
**do**
3:  Event-related data packet generated4:  **while** GW is out of one-hop range **do**5:   $\mathrm{Determine}\;\mathrm{the}\;\mathrm{candidate}\;\mathrm{relay}\;\mathrm{set}\;{\mathbb{N}}^{cf}$
$\mathrm{and}\;\mathrm{calculate}\;\mathrm{RFRV}\;\mathrm{for}\;\mathrm{each}\;EN\in {\mathbb{N}}^{cf}$
by Equation (4)6:   
$\mathrm{if}\;{\mathbb{N}}^{cf}=\emptyset \;$
**then**
7:    Go to line 168:**   end if**9:   Formulate state vector s
by Equation (16)10:   Get Q-values for s
in the evaluation network11:   Select action a
 via annealing ε-greedy
strategy12:   Forward data packet according to the action
 a
13:   Perform lines 16–2514:**  end while**15:  Send the data packet to the GW16:  Compute the reward r
by Equation (17)17:  State transitions to
$\;s^{\prime }$
18:  
$\mathrm{Store}\;\left(\bar{s},a,r,{\bar{s}}^{\prime }\right)$
 into M
$\;\mathrm{and}\;\mathrm{set}\;N_{\exp}=N_{\exp}+1$
19:  
$\mathrm{if}\;\left|M\right|\geq B$
$\;\mathrm{and}\;\mathrm{mod}\left(N_{\exp},C_{\exp}\right)=0\;$
**then**
20:   
$\mathrm{Sample}\;a\;\mathrm{random}\;\mathrm{minibatch}\;\left(s,a,r,s^{\prime }\right)$
 from M
21:   
$\mathrm{Update}\;\mathrm{target}\;Q-\mathrm{values}\;\mathrm{of}\;\mathrm{all}\;\mathrm{samples}\;\mathrm{with}\;y\left(s^{\prime },r\right)=\left\{\begin{array}{cc} r+\gamma \underset{a^{\prime }\in A}{\max}Q\left(s^{\prime },a^{\prime };\omega^{-}\right), & C_{3}\\ r, & otherwise \end{array}\right.$
22:   $\mathrm{Compute}\;\bar{L}\left(\omega \right)$
by Equation (2)23:   Update ω
in the evaluation network using SGD24:**  end if**25:  
$\mathrm{if}\;\mathrm{mod}\left(N_{\exp},C_{t}\right)=0$
$\;\mathrm{then}\;\omega^{-}=\omega$
26: 
**end for**
27:**end for****Output:** Evaluation network with ***ω***
**Online Decision****Input:** Trained evaluation network with ***ω***
28:DEN determines the set of candidate next-hop ENs and calculates RFRV for each candidate according to Equation (4)29:DEN constructs a state vector30:Input the current state into the evaluation network, and output the optimal action with maximum Q-value31:Forward the data packet according to the optimal action

## 6. Simulation Results and Analysis

In this section, we conduct extensive experiments to evaluate the performance of FRDR using MATLAB R2020b.

### 6.1. Simulation Models

It is assumed that each EN sends packets to the GW either directly or via intermediate hops using the LoRa protocol. The spreading factor (SF), bandwidth (BW), and coding rate $R_{c}$ are fixed at SF = 7, BW = 125 kHz, and $R_{c}=1$, respectively. The low data rate optimization is not enabled by default, while explicit header type (H = 0) and CRC are adopted. These parameter settings determine the simulation models adopted in the experiments, including those for packet transmission time, energy consumption, path loss, etc.

#### 6.1.1. Packet Transmission Time Measurement Model

The time for ENs to transmit a packet, ToA, is computed as follows [32]:

(27) $$ToA=\left\{\left(n_{pr}+4.25\right)+8+P_{1}\right\}\cdot 2^{SF}/BW,$$

(28) $$P_{1}=\max\left(\left\lceil \frac{8n_{pl}-4SF+28+16CRC-20H}{4\cdot SF}\right\rceil \cdot \left(R_{c}+4\right),0\right),$$

where $n_{pr}$ and $n_{pl}$ denote the symbol number of the preamble and payload, respectively.

#### 6.1.2. Energy Consumption Measurement Model

Given that energy consumption in the dormant state is significantly lower than that in other transceiver states, the energy consumption of an EN, denoted as $E_{c}$, can be simplified as the sum of transmit and receive energies [2]:

(29) $$E_{c}=V_{\mathrm{DD}}\cdot \left(I_{tx}\cdot T_{tx}+I_{rx}\cdot T_{rx}\right),$$

where $V_{\mathrm{DD}}$ is the nominal voltage, $I_{tx}$ and $I_{rx}$ denote the transmitting and receiving currents, respectively. $T_{tx}$ and $T_{rx}$ indicate the duration of transmitting and receiving, satisfying $T_{tx}=T_{rx}=ToA$. According to [33], these parameters are configured as specified in Table 5. Additionally, $P_{T}$ denotes the transmit power at the transmission power level $l_{tx}$.

#### 6.1.3. Path Loss Measurement Model

Results presented in this study were computed at carrier frequency f=868 MHz and the path loss model defined in [33]:

(30) $$\begin{array}{c} PL\left[\mathrm{dB}\right]=32.45+30\left(e_{pl}-2\right)+20\mathrm{lg}\left(f\left[\mathrm{MHz}\right]\right)\\ +e_{pl}\cdot 10\mathrm{lg}\left(d\left[\mathrm{km}\right]\right)+\delta [\mathrm{dB}] \end{array},$$

where PL is the path loss at distance d, $e_{pl}$ is the path loss index, and $\delta ∼Ν\left(0,\sigma^{2}\right)$ models random channel fluctuations resulting from shadowing. In this study, we use $e_{pl}=5$ and σ=3 dB.

#### 6.1.4. RSSI and SNR Measurement Models

Based on Equation (30), RSSI is computed as a function of $P_{T}$:

(31) $$RSSI\left[\mathrm{dBm}\right]=P_{T}\left[\mathrm{dBm}\right]+G_{A}^{T}\left[\mathrm{dB}\right]+G_{A}^{R}\left[\mathrm{dB}\right]-PL\left[\mathrm{dB}\right],$$

where $G_{A}^{T}$ and $G_{A}^{R}$ denote the transmitting and receiving antenna gains, respectively. In this study, we set $G_{A}^{T}=G_{A}^{R}=3\;\mathrm{dBi}$.

During data transmission, the noise power $P_{n}$ of each EN is considered in the calculation of SNR [29].

(32) $$SNR\left[\mathrm{dB}\right]=P_{r}\left[\mathrm{dBm}\right]-P_{n}\left[\mathrm{dBm}\right],$$

(33) $$P_{n}\left[\mathrm{dBm}\right]=10\mathrm{lg}\left(\left(T_{r}+T_{b}\right)\cdot BW\cdot \kappa \right)+30,$$

where $T_{b}$ is the background temperature, typically set to 290 K, $\kappa =1.379\times 10^{-23}\;J\cdot K^{-1}$ is the Boltzmann Constant, and $T_{r}$ is the receiver temperature, given by the following expression:

(34) $$T_{r}=(10^{NF/10}-1)\cdot T_{b},$$

where NF is the noise figure of the receiver. According to [29], the parameters are set as NF=6 dB, $RSSI_{th}=-124.5\;\mathrm{dBm}$, and $SNR_{th}=-7.5\;\mathrm{dB}$.

### 6.2. Simulation Setup

In this section, a comparison among FRDR, Minimum Hop Routing (MHR), and DQIR [15] is performed. MHR operates as a distributed routing algorithm that selects relays based on the minimum hop count to the GW. In DQIR, next-hop selection from candidate routers is conducted by a DQN-based routing protocol. Notably, to achieve a more equitable comparison, the learning rate and replay memory in DQIR are adjusted to 0.01 and 5000, respectively, through extensive hyperparameter tuning.

To comprehensively evaluate the performance of FRDR, we introduce three self-contrasting algorithms detailed in Table 6.

Additionally, to ensure fair benchmarking, all compared algorithms adopt the same pre-selection rules as FRDR and utilize the SPIN-based interaction process to determine candidate routers.

The dataset generation framework introduced in [34] is applied to deploy ENs in a stochastic and nonuniform manner across a 1 km×1 km area, while a GW is located at the center. The initial energy of all ENs is fixed at 0.5 mAh. During each iteration, a source EN is randomly selected from ENs, and a data packet is transmitted from this source EN to the GW under different multi-hop routing protocols. Additional network parameters are detailed in Table 7.

During the offline training of DQN, we execute 100 training episodes, and each episode comprises 1000 complete packet transmission simulations. For each simulation, a source EN is randomly chosen from the monitoring area, which then transmits a data packet toward the GW via single-hop or multi-hop routing. Upon transmission completion (either successful delivery to the GW or failure), the simulation proceeds immediately to the next packet transmission. Other specific DQN parameters are detailed in Table 8.

### 6.3. Performance Analysis

The performance metrics employed in our simulation are packet delivery rate (PDR), mean transmission delay (MTD), mean number of transmission hops (MTHs), mean energy consumption for delivering a data packet (MECP), and network lifetime. The network lifetime can be commonly measured in terms of the first node dead (FND), half node dead (HND), and the last node dead (LND). However, only HND is adopted in our simulation as the network was disabled long before LND, while the death of the first EN had little impact on network performance.

**Figure 5:**
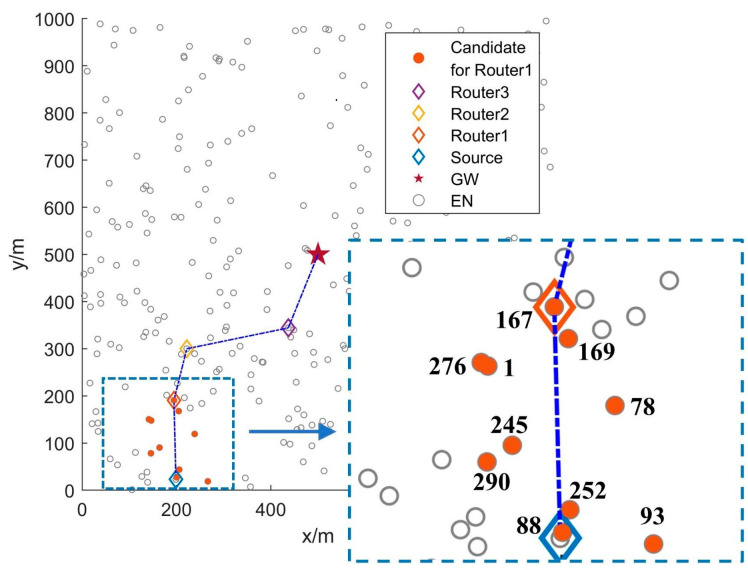
Simulation experiment scenario diagram of FRDR.

We randomly select an experimental scenario with 300 ENs from our simulations and use the routing process of the first data packet after network deployment to visually demonstrate the FRDR protocol in Figure 5. To clarify the relay selection mechanism of FRDR, Figure 5 further illustrates the spatial distribution of candidate ENs for Router1. Specifically, the source EN first adjusts its transmission power level according to PRM, thereby optimizing the EN activation range based on the average SNR of received signals from neighbors. In this case, the source EN broadcasts an ADV packet at Level 6. Subsequently, ENs that receive the ADV packet and satisfy pre-selection rules form the candidate set for Router1. Table 9 quantitatively summarizes their critical attributes, including ID, RFRV, distance to the GW, and residual energy. Then, by utilizing the DQN-based routing decision mechanism (Algorithm 3), the source EN determines Router1 from the candidates. Experimental result indicates that EN167, the candidate EN exhibiting the lowest RFRV and shortest distance to the GW, is selected as Router1. This outcome is consistent with the relay selection objective of FRDR defined in Equation (14).

**Figure 6:**
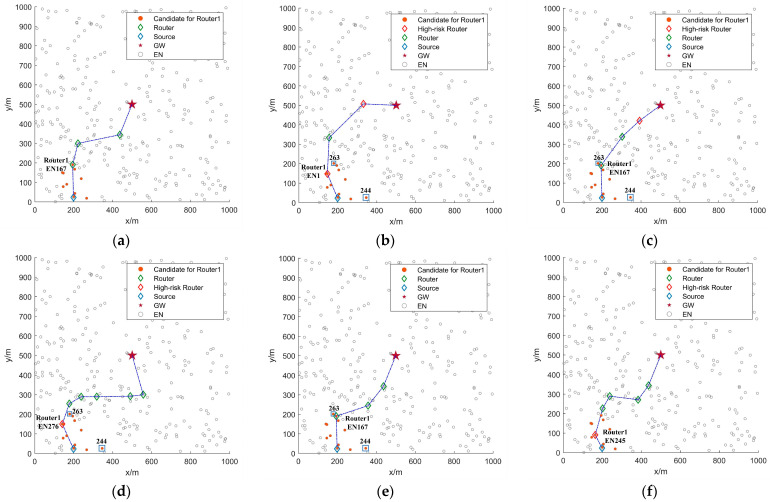
Simulation experiment scenario diagram of multi-hop routing: (**a**) FRDR, (**b**) MHR, (**c**) DQIR, (**d**) PFRS, (**e**) PFRD, and (**f**) PRRS.

With the identical configuration as Figure 5, Figure 6 further compares routing processes across different multi-hop routing algorithms. As shown in Figure 6, both FRDR and PRRS exhibit fewer candidate ENs for Router1. This reduction is primarily attributed to the PRM, which adjusts EN activation ranges based on the average SNR of received signals from neighbors. By utilizing PRM, the source EN in FRDR and PRRS reduces transmission power to Level 6, thereby confining the ADV broadcast range. In contrast, non-PRM protocols broadcast ADV packets at maximum power (Level 7), which can easily cause redundant EN activation (e.g., EN263 and EN244). Following candidate screening, each algorithm applies distinct criteria for final relay selection. Notably, PFRS and PRRS exhibit higher hop counts due to random relay selection, whereas FRDR, MHR, DQIR, and PFRD achieve lower hop counts through effective selection rules. Specifically, MHR selects relays via pre-stored routing tables guided by minimum hop counts, while DQIR prioritizes relays that minimize distance to the GW and balance residual energy distribution. FRDR and PFRD incorporate multi-dimensional neighborhood state characteristics of candidate ENs into routing decisions, effectively avoiding relays that introduce high routing failure risk (e.g., Router1 in MHR and Router3 in DQIR).

First, the performance analysis of the proposed PRM and Algorithm 3 is provided.

**Figure 7:**
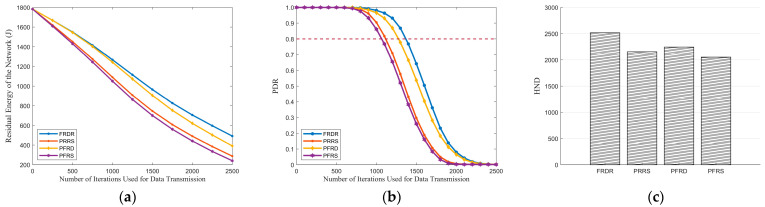
Comparisons between FRDR, PRRS, PFRD, and PFRS in terms of the residual energy of the network, PDR, and HND: (**a**) residual energy of the network, (**b**) PDR, and (**c**) HND.

Figure 7a demonstrates that PRM effectively reduces energy consumption. By employing PRM, DENs in FRDR and PRRS adjust transmission power levels according to demand, thereby reducing redundant EN activations. Consequently, the lifetime of individual EN can be extended, which in turn enhances the overall network lifetime and PDR. Figure 7b further illustrates that the PDR curves of PRRS and FRDR decline more slowly than PFRS and PFRD. Specifically, when the PDR of PFRD and PFRS drops to 0.80, FRDR and PRRS maintain values of 0.88 and 0.85, representing improvements of 10.00% and 6.25%, respectively. Additionally, as depicted in Figure 7c, both FRDR and PRRS achieve higher HND than their respective counterparts, which confirms the effectiveness of PRM in extending network lifetime.

Table 10 presents a comparison of MTH, MTD, and MECP for delivering the first 1000 packets, during which the PDR of each algorithm remains at a relatively high level. It reveals that PRM introduces a slight increase in transmission delay. Compared to PFRD and PFRS, the MTH of FRDR and PRRS increased by 0.20 and 0.30, respectively. This increase is attributed to the fact that the execution of PRM prevents the single-hop range from consistently reaching its maximum, potentially increasing the number of hops required for data delivery. Nevertheless, through the effective combination with pre-selection rules, PRM further amplifies the benefits of reducing redundant EN activations, thereby significantly decreasing the delay and energy consumption associated with REQ packet reception. As a result, the impact of these additional hops on overall delay is minimal. Specifically, the 0.14 s increase in MTD for FRDR constitutes only 3.26% of its total MTD, while for PRRS, it accounts for 3.13%. Overall, PRM achieves an effective balance between transmission efficiency and other critical performance metrics, including energy consumption, PDR, and network lifetime.

As for Algorithm 3, Figure 7b clearly illustrates its superiority in PDR. Specifically, when the PDR of PRRS and PFRS decreases to 0.80, FRDR and PFRD sustain values of 0.96 and 0.94, achieving improvements of 20.00% and 17.5%. These improvements arise from the multi-factor routing strategy of Algorithm 3, where RFRV, distance to the GW, transmission hops, and residual energy are considered in tandem. Therefore, Algorithm 3 effectively reduces transmission disruption and enables faster delivery to the GW while balancing energy consumption. The results presented in Figure 7c and Table 10 further confirm the advantage of Algorithm 3. In terms of HND, FRDR improved by 16.81%, while PFRD achieved a growth rate of 9.46%. Additionally, during the whole network lifetime, FRDR achieves reductions of 21.48%, 25.35%, and 24.64% in MTH, MTD, and MECP, respectively, compared to PRRS, while PFRD demonstrates reductions of 20.75%, 25.45%, and 26.03%.

Second, to fully illustrate the superiority of FRDR, a comparison among FRDR, MHR, and DQIR is presented.

**Figure 8:**
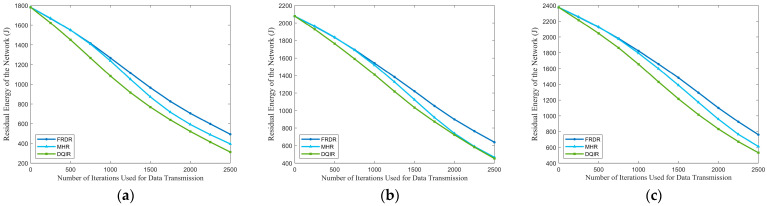
Residual energy of the network under different densities: (**a**) 300, (**b**) 350, and (**c**) 400.

**Figure 9:**
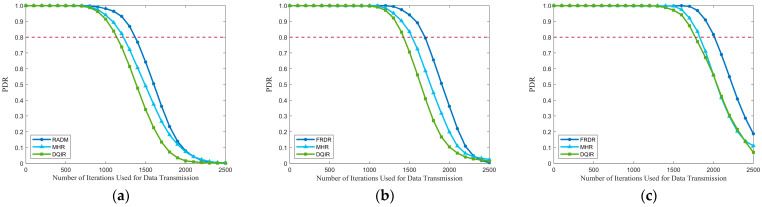
PDR under different densities: (**a**) 300, (**b**) 350, and (**c**) 400.

The comparisons of PDR and residual energy of the network among different EN densities are depicted in Figure 8 and Figure 9. It is evident that FRDR significantly outperforms MHR and DQIR by maintaining a higher PDR and reducing energy consumption. Moreover, Table 11 provides a comparison of network lifetime, while a more detailed comparison of MTH, MTD, and MECP across different EN densities is presented in Table 12. They demonstrate that FRDR achieves the longest network lifetime while maintaining comparable transmission delay. These improvements are attributed to the integration of PRM and the DQN-based multi-factor routing strategy, which dynamically adjusts activation range to reduce redundant activation and optimizes routing decisions based on RFRV, residual energy, transmission hops, and the distance to the GW.

MHR focuses solely on minimizing transmission hops, which contributes to its superiority in MTH, as shown in Table 12. However, to achieve this goal, the maximum transmission power is fixed in MHR, which leads to higher redundant activation than FRDR, particularly as EN density increases. This increased redundancy diminishes the delay and energy efficiency advantages gained by minimizing transmission hops, as higher reception delay and energy consumption occur during REQ reception. Table 12 further reveals that MHR results in a higher MECP than FRDR, while achieving a marginal reduction in MTD. Moreover, the exclusive consideration of hop count in MHR inevitably leads to hotspot issues due to the overutilization of partial ENs, which in turn leads to a shorter network lifetime and lower PDR. Conversely, by integrating RFRV and residual energy into routing decisions, FRDR effectively avoids routers that will introduce high routing failure risk and realizes a more balanced energy distribution. As a result, FRDR achieves a higher network lifetime and PDR. Specifically, when the PDR of MHR drops to 0.80, FRDR maintains a higher PDR, achieving improvements of 14.71%, 15.69%, and 18.90% at EN densities of 300, 350, and 400, respectively. Consequently, compared to MHR, FRDR effectively improves the PDR and network lifetime while maintaining a comparable transmission delay.

As for DQIR, multiple factors, including residual energy and distance to the GW, are considered when selecting the next-hop router from candidate ENs to minimize delay and balance energy distribution. Table 12 indicates that DQIR achieves lower MTH at EN densities of 350 and 400 compared to FRDR. However, DQIR requires all ENs that receive broadcast information to transmit a message to a designated agent for routing decisions. Although this method offloads the reception energy consumption from DEN to an additional agent without energy constraints, leading to more balanced energy consumption, the excessive overhead from replies significantly increases energy consumption and delay. In contrast, through the combination of PRM and pre-selection rules, FRDR effectively reduces redundant transmissions by dynamically adjusting activation ranges and requiring only ENs that meet the pre-selection rules to respond. As a result, compared to DQIR, FRDR achieves lower MTD and MECP, as well as a higher network lifetime. Moreover, by considering RFRV, FRDR effectively avoids selecting candidate routers that will introduce high routing failure risk, which further enhances the performance of PDR. Specifically, when the PDR of DQIR drops to 0.80, FRDR maintains a higher PDR, achieving improvements of 18.90%, 20.68%, and 21.96% at EN densities of 300, 350, and 400, respectively.

To summarize, the performance superiority of FRDR mainly comes from the PRM and DQN-based routing decision mechanism. PRM dynamically adjusts activation ranges, which works with pre-selection rules, further reducing unnecessary reception overhead. Meanwhile, the RFRV, in conjunction with other factors such as residual energy, distance to the GW, and transmission hops, is integrated into the DQN-based routing decision mechanism, effectively reducing transmission disruption and enabling faster delivery to the GW while balancing energy consumption. Consequently, FRDR significantly enhances PDR and network lifetime while maintaining a comparable transmission delay.

## 7. Conclusions

In this paper, we proposed a novel multi-hop routing protocol for LPWANs, named FRDR, which aims to reduce transmission disruption probability. FRDR comprehensively considered RFRV, distance to the GW, residual energy, and transmission hops as routing criteria, thereby deriving a low-latency, long-lifetime, and high-success-rate routing decision policy through a DQN-based framework. Simulation results confirmed that, compared with MHR and DQIR, our FRDR significantly reduces transmission disruption probability and extends network lifetime while maintaining a comparable delay. Specifically, when the PDR of MHR and DQIR drops to 0.80, FRDR maintains a higher PDR, achieving a minimum improvement of 14.71% and 18.90%, respectively.

Our current research focuses on multi-hop routing optimization under standardized scenarios with generalized assumptions. However, real-world deployments introduce non-ideal factors such as edge-positioned GWs, mobile GWs, and asymmetric link conditions, which significantly impact protocol performance. Consequently, future study will focus on addressing challenges arising from these non-ideal factors to enhance the robustness and scalability of the routing protocol in practical deployments. Additionally, field trials will be conducted to evaluate the practical feasibility and performance of the proposed protocol.

## Figures and Tables

**Table 1:** 1–9 scale.

Scale	Numerical Rating	Reciprocal
Equally importance	1	1
Slight importance	2	1/2
Moderate importance	3	1/3
Moderate to strong importance	4	1/4
Strong importance	5	1/5
Strong to very strong importance	6	1/6
Very strong importance	7	1/7
Very strong to extreme importance	8	1/8
Extreme importance	9	1/9

**Table 2:** Random consistency index.

*k*	1	2	3	4	5	6	7	8	9	10
RI	0	0	0.58	0.9	1.12	1.24	1.32	1.41	1.45	1.49

**Table 3:** Pairwise comparison matrix for three criteria in RFRV.

	$E_{n}$	$N_{n}$	LQ
$E_{n}$	1	2	3
$N_{n}$	1/2	1	2
LQ	1/3	1/2	1

**Table 4:** Pairwise comparison matrix for three criteria in ${\hat{r}}_{t}$.

	${\tilde{r}}_{t}^{1}$	${\tilde{r}}_{t}^{2}$	${\tilde{r}}_{t}^{3}$
${\tilde{r}}_{t}^{1}$	1	2	4
${\tilde{r}}_{t}^{2}$	1/2	1	3
${\tilde{r}}_{t}^{3}$	1/4	1/3	1

**Table 5:** Energy consumption parameters.

$l_{tx}$	7	6	5	4	3	2	1
$P_{T}$ [dBm]	14	12	10	8	6	4	2
$I_{tx}$ [mA]	38	35.1	32.4	30	27.5	24.7	22.3
$I_{rx}$ [mA]	14.2						
$V_{\mathrm{DD}}$ [V]	3.3						

**Table 6:** Self-contrasting algorithms.

	Transmit Power Level	Routing Decision Mechanism
PFRS	$\mathrm{Transmit}\;\mathrm{with}\;l_{tx}^{\max}$	Random selection
PFRD	$\mathrm{Transmit}\;\mathrm{with}\;l_{tx}^{\max}$	Algorithm 3
PRRS	PRM	Random selection
FRDR	PRM	Algorithm 3

**Table 7:** Network parameters.

Parameters	Value	Parameters	Value
N	300/350/400	$e_{th}$	100 mJ
$R_{change}^{-}$	0.5	$n_{pr}$	8 symbols
$n_{pl}$ of ADV/REQ	1 B	$n_{pl}$ of data packet	300 B

**Table 8:** Parameters of DQN.

Parameters	Value	Parameters	Value
$N_{eps}^{max}$	100	$N_{iter}^{max}$	1000
α	0.009	γ	0.95
$\left|M\right|$	5000	B	64
$C_{\exp}$	10	$C_{t}$	400
$R_{\max}$	1	τ	0.2
η	2	$H_{\max}$	10
$\varepsilon_{start}$	0.5	$\varepsilon_{end}$	0.01

**Table 9:** Critical attributes of candidate EN for Router1.

ID	RFRV	Distance (m)	Residual Energy (J)
1	0.1648	499.0684	5.9358
78	0.3042	461.9026	5.9358
88	0.2711	559.5603	5.9358
93	0.4604	534.8417	5.9358
167	0.1247	436.6732	5.9358
169	0.1389	444.5297	5.9358
245	0.2584	529.7191	5.9358
252	0.3133	542.9591	5.9358
276	0.2191	500.7071	5.9358
290	0.2280	550.7258	5.9358

**Table 10:** MTH, MTD, and MECP for delivering the first 1000 packets.

	PFRS	PRRS	PFRD	FRDR
MTH	4.82	5.12	3.82	4.02
MTD (s)	5.58	5.76	4.16	4.30
MECP (J)	0.73	0.69	0.54	0.52

**Table 11:** HND under different densities.

	FRDR	MHR	DQIR
300	2516	2249	2205
350	2651	2298	2445
400	2736	2420	2452

**Table 12:** MTH, MTD, and MECP for delivering the first 1000 packets under different densities.

		FRDR	MHR	DQIR
300	MTH	4.02	3.76	4.06
	MTD (s)	4.30	4.14	5.94
	MECP (J)	0.52	0.54	0.70
350	MTH	3.88	3.57	3.60
	MTD (s)	4.30	4.07	5.47
	MECP (J)	0.54	0.56	0.67
400	MTH	3.65	3.39	3.52
	MTD (s)	4.21	4.04	5.72
	MECP (J)	0.55	0.58	0.72

## Data Availability

The original contributions presented in this study are included in the article. Further inquiries can be directed to the corresponding author.

## Abbreviations

The following abbreviations are used in this manuscript:

ADR	Adaptive Data Rate algorithm
ADV	Advertisement
AHP	Analytical Hierarchy Process
BW	Bandwidth
CI	Consistency Index
CR	Consistency Ratio
DEN	Data-holding End-device Node
DNN	Deep Neural Network
DQN	Deep Q-Network
DQIR	DQN-based Intelligent Routing protocol
EN	End-device Node
FND	First Node Dead
FRDR	Failure Risk-aware Deep Q-network-based multi-hop routing protocol
GPS	Global Positioning System
GW	gateway
HND	Half Node Dead
LND	Last Node Dead
LPWAN	Low-Power Wide-Area Network
MDP	Markov Decision Process
MECP	Mean Energy Consumption for Delivering a Data Packet
MHR	Minimum Hop Routing protocol
MTD	Mean Transmission Delay
MTH	Mean Number of Transmission Hops
PDR	Packet Delivery Rate
PRM	Power Regulation Mechanism
REQ	Request
RFRV	Routing Failure Risk Value
RI	Random Consistency Index
RL	Reinforcement Learning
RSSI	Received Signal Strength Indicator
SF	Spreading Factor
SGD	Stochastic Gradient Descent
SNR	Signal-to-Noise Ratio
SPIN	Sensor Protocol for Information via Negotiation
